# Effect of the BTK inhibitor ibrutinib on macrophage- and γδ T cell-mediated response against *Mycobacterium tuberculosis*

**DOI:** 10.1038/s41408-018-0136-x

**Published:** 2018-11-05

**Authors:** Ana Colado, Melanie Genoula, Céline Cougoule, José L. Marín Franco, María B. Almejún, Denise Risnik, Denise Kviatcovsky, Enrique Podaza, Esteban E. Elías, Federico Fuentes, Isabelle Maridonneau-Parini, Fernando R. Bezares, Horacio Fernandez Grecco, María Cabrejo, Carolina Jancic, María del Carmen Sasiain, Mirta Giordano, Romina Gamberale, Luciana Balboa, Mercedes Borge

**Affiliations:** 1 Laboratorio de Inmunología Oncológica, Instituto de Medicina Experimental (IMEX)-CONICET-Academia Nacional de Medicina (ANM), CABA, Argentina; 2 Laboratorio de Inmunología de Enfermedades Respiratorias, IMEX-CONICET-ANM, CABA, Argentina; 3 International Associated Laboratory (LIA) CNRS/CONICET “IM-TB/HIV” (1167), Toulouse, France; 4 International Associated Laboratory (LIA) CNRS/CONICET “IM-TB/HIV” (1167), Buenos Aires, Argentina; 5 Institut de Pharmacologie et de Biologie Structurale, IPBS, Université de Toulouse, CNRS, UPS, Toulouse, France; 6 Departamento de Fisiología, Biología Molecular y Celular, Facultad de Ciencias Exactas y Naturales, Universidad de Buenos Aires, CABA, Argentina; 7 IMEX-CONICET-ANM, CABA, Argentina; 8 Sección de Hematología, Hospital General de Agudos Dr. Teodoro Álvarez, CABA, Argentina; 9 Departamento de Hematología, Sanatorio Julio Méndez, CABA, Argentina; 10 Laboratorio de Inmunidad Innata, MEX-CONICET-ANM, CABA, Argentina; 11 Departamento de Microbiología, Parasitología e Inmunología, Facultad de Medicina, Universidad de Buenos Aires, CABA, Argentina

The Bruton’s tyrosine kinase (BTK) inhibitor ibrutinib is approved by the Food and Drug Administration for its use as first-line treatment in chronic lymphocytic leukemia (CLL). Despite its efficacy, patients treated with ibrutinib rarely achieve complete responses and usually remain under treatment until progression. Considering the inherent high susceptibility of CLL patients to infections, a better understanding of ibrutinib effects on the immune system might help to estimate the risk of infectious complications on treated patients. Besides its effects on leukemic B cells, ibrutinib also affects functions on T cells, natural killer cells, and macrophages^1–3^. Macrophages are central players of the innate immune response against fungi, extracellular bacteria, and in particular against the intracellular bacteria *Mycobacterium tuberculosis* (*Mtb*). The World Health Organization (WHO) estimates that one-third of the world’s population is infected with *Mtb*, the causing agent of tuberculosis, a severe infection that kills near 1.3 million people per year and the leading cause of death from a single infectious agent (Global TB report, WHO, 2017). Notably, the incidence rate of tuberculosis is highly variable among different countries. In South American countries, such as Argentina and Brazil, where ibrutinib is being introduced, the rates of tuberculosis incidence are up to 14 times higher than in USA and other developed countries. Given the relevance of macrophages in *Mtb* immune response, we here evaluated the in vitro effects of ibrutinib on this cell compartment. Additionally, we studied its effects on γδ T cells, another innate immune component reported to be involved in *Mtb* response^4^. Strikingly, we found that ibrutinib affects macrophage's phenotype and the response of both macrophages and γδ T cells to *Mtb*.

Macrophages were differentiated from human monocytes with macrophage colony-stimulating factor (M-CSF), then pre-treated with ibrutinib for 30 min, and afterward exposed to irradiated *Mtb* for 24 h. We found that clinically relevant doses of ibrutinib (0.03–0.3 µM) significantly reduced the release of tumor necrosis factor (TNF)-α (Fig. 1a and Supplementary Fig. 1a), while interleukin (IL)-10 and IL-8 secretion was only affected at 3 µM, which is a concentration higher than the one reported in the plasma of treated patients (Fig. 1b, c). Importantly, macrophage viability was not affected by ibrutinib (Supplementary Fig. 1b). Inhibition of TNF-α secretion was associated with a diminished phosphorylation of the p65 subunit of the transcription factor nuclear factor (NF)-kB (Fig. 1d), a key regulator of cytokine production in macrophages. Given the relevance of Toll-like receptor (TLR) 2 and TLR4 in *Mtb* recognition by macrophages, we evaluated the effect of ibrutinib on TNF-α secretion in response to lipopolysaccharide (LPS), a TLR4 ligand, and Pam3CSK4, a TLR2 ligand. Results in Fig. 1e show that ibrutinib impaired TNF-α secretion induced by these ligands. Of note, the inhibition in response to TLR2, but not to TLR4, stimulation was observed even at low concentrations of ibrutinib (30 nM) (Supplementary Fig. 1c, d), suggesting a differential involvement of BTK, or other ibrutinib targets, in TLR2 and TLR4 signaling pathways. Inhibition of TNF-α secretion in response to *Mtb*, Pam3CSK4, and LPS was also observed in ibrutinib-treated monocyte-derived macrophages from CLL patients (Fig. 1f). Clinical characteristics of CLL patients included in this study are presented in Supplementary Table 1.

**Fig. 1:**
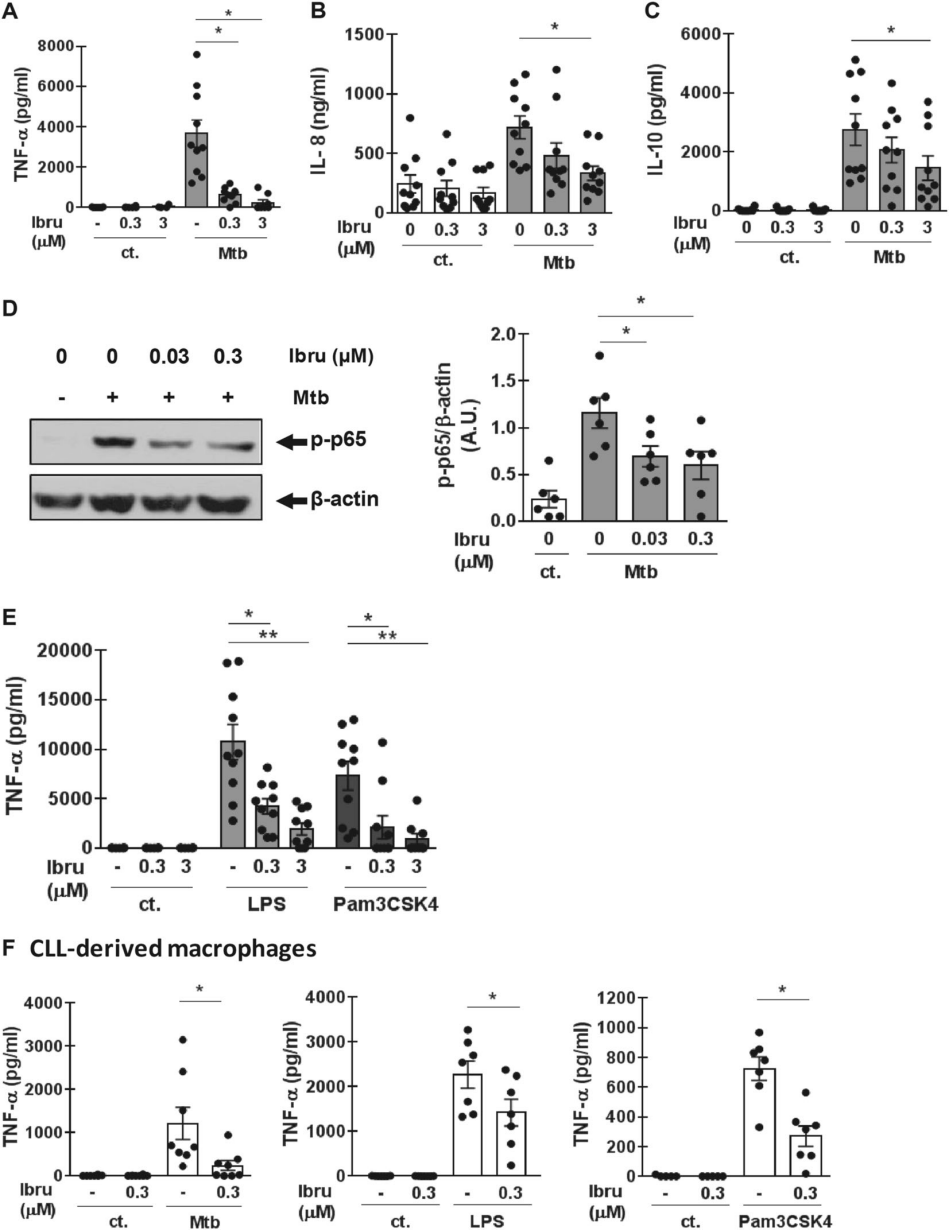
Ibrutinib impairs macrophage-mediated response against *Mycobacterium tuberculosis*. Macrophages were obtained by culturing monocytes from healthy donors (HD) or CLL patients for 5 days in RPMI 10% FCS in the presence of M-CSF (50 ng/ml). **a**–**c** HD-macrophages were stimulated with irradiated *Mtb* (MOI equivalent to 2) in the presence or absence of ibrutinib (Ibru) and after 24 h TNF-α, IL-8, and IL-10 secretion was measured by ELISA in culture supernatants. Bars represent mean ± SEM of cytokine concentration in control (ct.) cultures (white bars) or *Mtb*-stimulated cultures (gray bars). *n* = 10, *p < 0.05, Kruskal–Wallis test, followed by Dunn’s multiple comparisons test. **d** Phosphorylation of p65 (p-p65) was evaluated by western blot in HD macrophages after 15 min of stimulation with *Mtb* in the presence or absence of ibrutinib. Bands on the immunoblots were quantified using the ImageJ software (NIH Image). Results are shown as the mean ± SEM of the ratio p-p65/β-actin in arbitrary units (A.U.). *n* = 6, *p < 0.05, Friedman test, followed by Dunn’s multiple comparisons test. **e** HD macrophages were stimulated with LPS (100 ng/ml) or Pam3CSK4 (100 ng/ml) in the presence or absence of ibrutinib for 24 h and TNF-α production was measured in culture supernatants by ELISA. *n* = 10, *p < 0.05, Kruskal–Wallis test, followed by Dunn’s multiple comparisons test. **f** CLL macrophages were stimulated with irradiated *Mtb* (MOI equivalent to 2), LPS (100 ng/ml), or Pam3CSK4 (100 ng/ml) in the presence or absence of ibrutinib for 24 h and TNF-α production was measured by ELISA in culture supernatants. *n* = 7, *p < 0.05, Kruskal–Wallis test, followed by Dunn’s multiple comparisons test

Then, given that macrophages with different polarization profiles have different abilities to promote an efficient immune response against *Mtb*, the M1 pro-inflammatory profile being more effective than the M2 anti-inflammatory profile^5^, we aimed to evaluate the effect of ibrutinib on macrophage polarization in interferon (IFN)-γ-induced M1, IL4-induced M2, and IL-10-induced M2 profiles. M1 and M2 polarization was confirmed by analyzing the expression of CD16, CD14, CD163, CD206, CD86, and HLA-DR by flow cytometry (Supplementary Fig. 2). We found that ibrutinib impaired M1 polarization as shown by the upregulation of M2-associated markers CD16, CD14, CD163, and CD206 and by downregulation of the M1-associated markers CD86 and HLA-DR (Fig. 2a, b). The expression of these markers was not modified by ibrutinib on IL-4- and IL-10-induced M2 macrophages (Supplementary Fig. 3). These results are in line with those recently published by Fiocari et al. showing that ibrutinib promotes an M2 phenotype in nurse-like cells from CLL patients^2^. Impairment in M1 polarization, induced upon ibrutinib exposure, was not associated with a decrease in signal transducer and activator of transcription factor (STAT) 1 phosphorylation (Supplementary Fig. 4). Interestingly, we found that ibrutinib enhanced macrophage three-dimensional migration in Matrigel (Fig. 2c), a feature of macrophage with an M2 profile^6^. We also found that M1 macrophages polarized in the presence of ibrutinib showed a decreased secretion of TNF-α and an increased secretion of IL-10 compared to control M1 macrophages, resembling the cytokine profile associated with M2 macrophages (Fig. 2d). M1 and M2 macrophages also differ in their glucose metabolic pathways. During M1 polarization, macrophages activate the aerobic glycolytic pathway, increasing glucose uptake and lactate production, while M2 macrophages preferentially use the oxidative metabolism to obtain energy. Importantly, this switch towards aerobic glycolysis seems to be necessary for an effective differentiation into the M1 profile^7^. We found that ibrutinib treatment of M1 macrophages reduced both glucose consumption and lactate production (Fig. 2e). The impairment in M1 polarization, and the reduction in glucose consumption and lactate production induced by ibrutinib were also confirmed in macrophages from CLL patients (Supplementary Fig. 5). We also evaluated whether ibrutinib affects the functionality of polarized macrophages and found that ibrutinib decreased TNF-α production in response to *Mtb* and increased migration in matrigel in M1 macrophages (Supplementary Fig. 6). Taken together, these results showed that ibrutinib affects M1 polarization of macrophage and their function, which could have detrimental consequences on the immune response to *Mtb* in patients treated with ibrutinib.

**Fig. 2:**
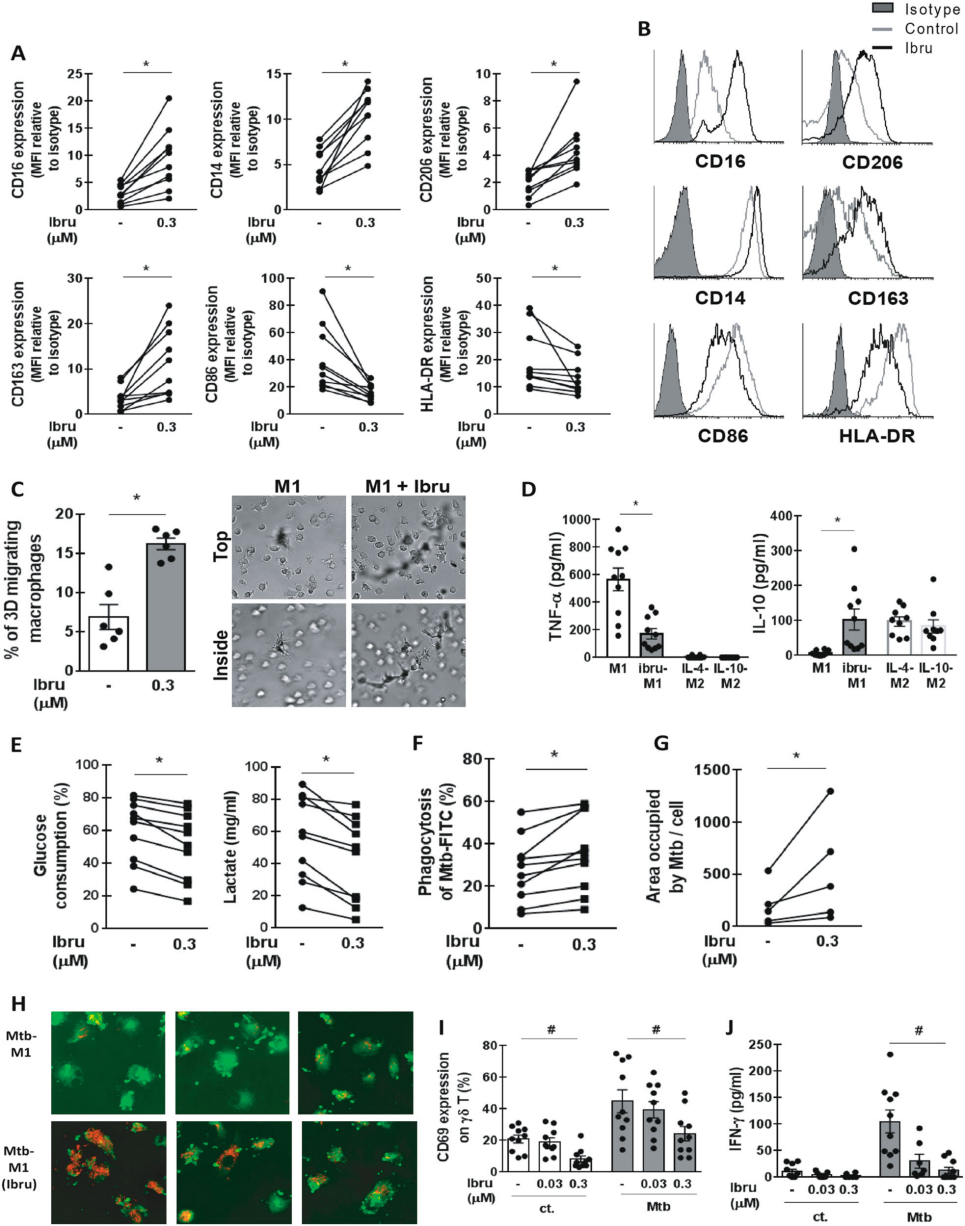
Ibrutinib impairs M1 polarization and affects macrophage and γδ T cell response to *Mycobacterium tuberculosis*. To obtain the M1 profile, monocytes from HD were cultured with GM-CSF (50 ng/ml) for 7 days and IFN-γ (10 ng/ml) was added for the last 2 days of culture. Ibrutinib (0.3 µM) was added to the culture 30 min before adding IFN-γ. **a** At day 7, macrophages were detached and stained with anti-CD14 PerCP/Cy5.5, anti-CD16 FITC, anti-CD86 PerCP/Cy5.5, anti-CD206 FITC, anti-CD163 PE, or anti-HLA-DR FITC and analyzed by using a FACScan flow cytometer (BD Immunocytometry Systems, San Jose, CA, USA). The results are shown as the mean fluorescence intensity (MFI) normalized to the MFI of the isotype control, *n* = 10. **b** Histograms of a representative experiment are shown. **c** M1 macrophages differentiated in the presence or absence of ibrutinib (0.3 µM) as described above were seeded on top of a thick layer of Matrigel in the upper Transwell chamber. The lower chamber was filled with medium with CCL5. Migration was quantified after 3 days. Results are shown as the percentage of migrated macrophages and representative images are shown in the right panel. Representatives images from the top and inside of matrigel are shown. **d** M1 macrophages were obtained as described above. M2 macrophages were obtained by culturing monocytes from healthy donors with M-CSF (50 ng/ml) for 7 days and either IL-4 (20 ng/ml) or IL-10 (10 ng/ml) were added during the last 2 days of culture. Ibru-M1 macrophages were treated with ibrutinib during the last 2 days of culture. At day 7, the medium was removed and fresh medium without cytokine was added. Then TNF-α and IL-10 was evaluated after 24 h of culture by ELISA. Bars show mean ± SEM. **e** M1 macrophages differentiated in the presence or absence of ibrutinib (0.3 µM) as described above. At day 7, glucose and lactate concentration in the supernatant was evaluated by using commercial kits. Glucose consumption was calculated as the percentage of glucose in the culture at day 7 relative to the initial glucose concentration in the medium. **f** M1 macrophages were differentiated in the presence or absence of ibrutinib as described before and stimulated with irradiated *Mtb*-FITC (MOI equivalent to 5) for 2 h, then cells were trypsinized and the percentage of FITC^+^ macrophages was analyzed by flow cytometry. Results are shown as the percentage of FITC^+^ macrophages, *n* = 10. **g**, **h** Monocytes from healthy donors were differentiated into M1 macrophages and were infected with the red fluorescent protein (RFP) expressing *M. tuberculosis* strain at an MOI of 5 during 2 h at 37 °C. Thereafter, ibrutinib at 0.3 µM or vehicle was added. After 48 h, the glass coverslips were fixed with PFA 4% and stained with BODIPY 493/503 (Life Technologies). Finally, slides were mounted and visualized with a FluoView FV1000 confocal microscope (Olympus, Tokyo, Japan) equipped with a Plapon ×60/NA1.42 objective and then analyzed with the software ImageJ-Fiji. **g** Quantification of the occupied area with RFP-*M. tuberculosis* (expressed as Raw Integrated Density) per cell in *z*-stacks from confocal laser scanning microscopic images. Individual cells were defined by BODIPY-stained cellular membranes that allow us to define the regions of interest for quantification. In all, 80–100 cells of random fields per condition were analyzed. **h** Representative microphotographs are shown. **i**, **j** Purified γδ T cells were stimulated with irradiated *Mtb* (MOI equivalent to 5) in the presence or absence of ibrutinib. After 24 h, CD69 expression and IFN-γ production were evaluated by flow cytometry and ELISA, respectively. **i** Results are shown as the percentage of γδ T cells expressing CD69. **j** IFN-γ concentration in the culture supernatant evaluated by ELISA. *p < 0.05, Wilcoxon matched-pairs signed rank test. ^#^*p* < 0.05, Kruskal–Wallis test, followed by Dunn’s multiple comparisons test

Then, since treatment of macrophages with ibrutinib during M1 polarization increased CD206 expression (Fig. 2a), a receptor involved in *Mtb* phagocytosis, we evaluated whether macrophage phagocytosis and/or the intracellular growth of *Mtb* was affected in this situation. As shown in Fig. 2f, M1 macrophages polarized in the presence of ibrutinib showed a slight increase in *Mtb* uptake, while the intracellular growth of the bacteria was not modified (Supplementary Fig. 7). On the other hand, when we compared the effect of ibrutinib on the intracellular growth of already infected M1 macrophages, we found that ibrutinib impaired their killing capacity as shown by the increase in the bacillary load (Fig. 2g, h).

T cells bearing the γδ T cell receptor also participate in the innate host defense against *Mtb*. γδ T cells are found in *Mtb*-induced lesions in humans, they release IFN-γ in response to *Mtb*, a cytokine associated with protective immunity, and are cytotoxic against *Mtb*-infected macrophages resulting in killing of intracellular bacilli^4^. Therefore, we evaluated the effect of ibrutinib on human γδ T cells stimulated with *Mtb* and found a significant decrease in the expression of the activation marker CD69 and in the secretion of IFN-γ (Fig. 2i, j), suggesting that γδ T cell response to *Mtb* might also be compromised in ibrutinib-treated patients.

Results presented here show that ibrutinib affects macrophages and γδ T cells, which are important players in an effective immune response to *Mtb*. In particular, we observed that ibrutinib, used at doses found in the plasma of treated patients (0.03–0.3 µM), impairs immune mechanisms that contribute to the control of *Mtb* infection such as TNF-α secretion by macrophages, M1 polarization, *Mtb* intracellular growth in M1 macrophages, and IFN-γ secretion by γδ T cells. Importantly, the ability of the host to limit *Mtb* at the site of infection depends on the formation and maintenance of an effective granuloma structure. In this context, TNF-α production by activated macrophages plays a key role in *Mtb* control by promoting granuloma formation. In fact, patients treated with TNF-α-blocking agents have an increased risk of tuberculosis and the assessment of latent infection is recommended before starting such treatment. Our results showing that ibrutinib significantly decrease TNF-α secretion in response to *Mtb* suggests that this mechanism might be compromised in ibrutinib-treated patients.

Although ibrutinib treatment in CLL is associated with lower rates of infections compared to the standard chemoimmunotherapy (Fludarabine, Cyclophosphamide, Rituximab), major infections, particularly those of the respiratory tract, are still significant, reported in about one-third of ibrutinib-treated patients^8–10^. The risk of infections appears to be highest during the first 6 months of treatment, and a subsequent improvement in patient's cellular immunity has been suggested by the observation that ibrutinib favors a Th1 polarization^1^ and an increase in the T cell repertory^11^. Also, recovery from a refractory state of monocytes and T cells, by the downregulation in programmed cell death protein 1/programmed cell death ligand 1 expression, was suggested in a study with a small cohort (*n* = 4) of ibrutinib-treated patients^12^. Nevertheless, over the past years, opportunistic infections during ibrutinib treatment such as invasive pulmonary aspergillosis, disseminated *fusarium* infection, and disseminated cryptococcal disease have been reported^13^, and remarkably in 2015, a case of a CLL patient who developed miliary tuberculosis 1 month after initiation of ibrutinib treatment was reported^14^. In most of those cases, patients did not present neutropenia and showed normal levels of T cells suggesting that other/s component/s of the immune system might be affected in ibrutinib-treated patients. Moreover, an association between ibrutinib treatment and an increased susceptibility to *Pneumocystis jirovecii* pneumonia (PCP) in CLL was suggested by Ahn et al. who showed a 5% of incidence of PCP in a cohort of 96 patients treated with ibrutinib as a single agent^15^. In this report, patients who developed PCP did not have a low CD4^+^ T cell count, which is considered the primary risk factor for PCP. Interestingly, alveolar macrophages, TLR2 signaling, and TNF-α secretion are involved in an effective immune response against *P. jirovecii*. Given that TLR2 is a receptor also involved in the recognition of motifs present in several fungi, our finding that ibrutinib impairs macrophages’ response through TLR2 might explain, at least in part, the increased susceptibility of ibrutinib-treated patients to PCP and also the presence of other opportunistic fungal infections.

Although further studies are needed to confirm the relevance of our observations in vivo and their impact in clinical practice, we consider that these results represent a warning especially in countries with a high incidence of tuberculosis.

## Electronic supplementary material

Supplementary Data, Table and Figures
