# Supplementary material for: Effect of the BTK inhibitor ibrutinib on macrophage- and γδ T cell-mediated response against Mycobacterium tuberculosis
Source: Blood Cancer J. 2018 Nov 5;8(11):100. doi: 10.1038/s41408-018-0136-x (PMC6218455; doi:10.1038/s41408-018-0136-x)
Supplement: Supplementary file 1 — Supplementary Data, Table and Figures [file 41408_2018_136_MOESM1_ESM.docx]

**Supplementary data**

**Materials and methods**

**Reagents and antibodies**

RPMI 1640 was purchased from Life Technologies (NY, USA) and penicillin and streptomycin were obtained from GIBCO Laboratories (NY, USA). Fetal calf serum (FCS) was purchased from Natocor (CBA, Argentina).

## [PerCP/Cy5.5anti-CD14 (clone HCD14), PE anti-CD14 (clone HCD14), FITC anti-CD16 (clone 3G8), PerCP/Cy5.5anti-CD86 (clone IT2.2), Alexa Fluor®488 anti-CD206 (clone 15-2), FITC anti-CD206 (clone C068C2), PE anti-CD163 (clone GHI/61) and FITC anti-HLA-DR (clone L243) m](https://www.biolegend.com/en-us/products/percp-cy5-5-anti-human-cd14-antibody-4253)Abs were obtained Biolegend (CA, USA). PE-Cy™5 anti-CD16 (clone 3G8), PE anti-CD69 (clone FN50), PE anti-CD86 (clone 2331), PerCP/Cy5.5 anti-CD163 (clone [GHI/61](https://eur01.safelinks.protection.outlook.com/?url=https://www.biolegend.com/en-us/search-results?Clone=GHI/61&data=02|01||230d3ff402a64bfc11ef08d55c29cc23|84df9e7fe9f640afb435aaaaaaaaaaaa|1|0|636516256940275567&sdata=wAiQw7exSQMYMnckvzD3f7vmJbrm8X/v6EzuC+Wu5b4=&reserved=0)) and FITC anti-HLA-DR (clone G46-6) mAbs were purchase from BD Bioscience, Pharmingen (CA, USA) and APC anti-MerTK(clone 125518) was obtained from R&D systems (MN, USA).

Recombinant human M-CSF, recombinant human IL-10 and IFN-γ were purchased from Biolegend. Human GM-CSF from MiltenyiBiotec (Germany). Recombinant human IL-4 was obtained from eBioscience (CA, USA).

Pam3CSK4 and lipopolysaccharide (LPS) from Escherichia coli O111:B4 were obtained from Sigma-Aldrich.(St. Louis, MO, USA)

Polyclonal antibody(pAb) specific for p-NFκB p65 (Ser 311) was purchased from Santa Cruz Biotechnology (Texas, USA). Monoclonal Ab (mAb) anti-β-actin (8H10D10) and anti-pY701-STAT1 (58-D6) were from Cell Signalling (MA, USA). The HRP-conjugated mAb for mouse IgG was purchased from Sigma-Aldrich and the HRP-conjugated mAb for rabbit IgG from Jackson Immuno Research, Inc (PA, USA).

For ELISA assays, anti-human TNF-α, IL-8, IL-10 and IFN-ɣ kits were purchased from Biolegend.

Middlebrook 7H9 broth and Middlebrook 7H11 agar, OADC and DMSO were obtained from Sigma-Aldrich. Ibrutinib was obtained from MedKooBiosciencie (NC, USA).

**CLL patients and healthy donor samples**

Peripheral blood samples were obtained from healthy donors (HD) and CLL patients. Informed consent was obtained from all participants included in the study in accordance with the Declaration of Helsinki and with Institutional Review Board approval from the Academia Nacional de Medicina, Buenos Aires. CLL was diagnosed according to standard clinical and laboratory criteria. At the time of the analysis all patients were free from clinically relevant infectious complications, and were either untreated or had not received treatment for a period of at least 6 months before investigation. Clinical characteristics of CLL patients included in the study are shown in Supplementary Table 1.

***Mycobacterium tuberculosis* strain**

The γ-irradiated Mycobacterium tuberculosis (*Mtb*) H37Rv strain (NR-49098)was obtained from BEI Resource (USA).For assays involving viable *Mtb*, *Mtb* H37Rv strain was grown at 37°C in Middlebrook 127 7H9 medium (Difco) supplemented with 10% albumin-dextrose-catalase (Difco) and 128 0.05% Tween-80 (Sigma-Aldrich). Infections were performed in the biosafety level 3 (BSL-3) laboratory at the Unidad 195 Operativa Centro de ContenciónBiológica (UOCCB), ANLIS-MALBRAN (Buenos 196 Aires), according to the biosafety institutional guidelines.

**Cell separation procedures and culture**

Peripheral blood mononuclear cells (PBMC) were isolated from HD or CLL patients fresh blood samples by centrifugation over a Ficoll-Paque layer (GE Healthcare, UK), washed twice with saline solution and resuspended in RPMI 1640 supplemented with 10% FCS, 100U/mL penicillin and 100 µg/mL streptomycin. HD monocytes were isolated from PBMC by using a Percoll density gradient (GE Healthcare, % purity >90) and CLL monocytes by using magnetic cell sorting with the anti-CD14 human MicroBeads isolation kit according to the manufacture’s recommendations (MiltenyiBiotec, Germany, % purity > 98, evaluated by CD11b and CD14 expression by flow cytometry). γδ T cells from HD were also purified from PBMC by using magnetic cell sorting with the anti-TCR γ/δ human MicroBead isolation kit (MiltenyiBiotec, % purity> 98).

**Macrophages cultures**

Macrophages were differentiated by culturing monocytes from HD or CLL patients in RPMI 1640 medium with 10 % FCS in presence of M-CSF (50 ng/ml) for five days. To obtain M1 profile, cells were cultured in 500 µL of RPMI 10 % FCS supplemented with GM-CSF (50 ng/ml) for five days and two additional days with GM-CSF + IFN-γ (10 ng/ml). For M2 profile, monocytes were culture 5 days in 500 µL of RPMI 10 % FCS supplemented with M-CSF (50 ng/ml) and two additional days with M-CSF + IL-4 (20 ng/ml) or M-CSF + IL-10 (10 ng/ml) to induce IL-4-M2 or IL-10-M2 polarization, respectively.

**Immunofluorescence analyses**

HD- M1, IL-4-M2 and IL-10-M2 macrophages were differentiated as described before and ibrutinib (0.3 µM) or the vehicle of the drug (DMSO) was added during the last 2 days before cytokine addition. Then macrophages were detached with cold PBS and stained with anti-CD14PerCP/Cy5.5, anti-CD16 FITC, anti-CD86 PerCP/Cy5.5, anti-CD206 FITC, anti-CD163 PE or anti-HLA-DR FITCand analyzed by using a FACScan flow cytometer (BD Immunocytometry Systems, San Jose, CA, USA).CLL-M1 macrophages were stained with anti-CD16PE-Cy™5, anti-CD14 PE, anti-CD86 PE, anti-CD206 Alexa Fluor®488, anti-CD163PerCP/Cy5.5 and anti-HLA-DR FITC and expression level of the markers was analyzed by using a FACScan flow cytometer (BD Immunocytometry Systems, San Jose, CA, USA).

**Macrophages metabolism**

HD- or CLL-M1 macrophages were differentiated as described previously and ibrutinib (0.3 µM) or the vehicle of the drug (DMSO) was added during the last 2 days before cytokine addition. At day 7 glucose consumption and lactate production were measured in culture supernatants by using glucose and lactate kits from Wiener Laboratories (code 1400060 and 1999795) (Santa Fé, Argentina). For glucose consumption culture medium alone was used as control.

**Phagocytosis assay**

HD- macrophages were differentiated into M1 profile as described previously and ibrutinib (0.3 µM) or the vehicle of the drug DMSO was added during the last 2 days before cytokine addition. Then irradiated *Mtb*-FITC (MOI equivalent to 5) was added to cultures. After 2 h cells were trypsinized and phagocytosis was determinated as the percentage of FITC^+^macrophages by flow cytometry.

**Colony-forming units assay**

M1 macrophages were differentiated in the presence or absence of ibrutinib (0.3 µM) or DMSO as described before. At day 7, cells were infected with *Mtb* (MOI= 2) during 1 h at 37ºC. Then, extracellular bacteria was removed gently by washing with pre-warmed PBS, and cells were cultured in RPMI-1640 medium supplemented with 10 % FBS and gentamicin (50 μg/ml). At days 3 and 6, cells were lysed in 0.1 % SDS and neutralized with 20 % Bovine Serum Albumine in Middlebrook 7H9 broth. Serial dilutions of the lysates were plated in triplicate, onto 7H11-Oleic Albumin Dextrose Catalase (OADC, Difco) agar medium for CFU scoring at 21 days later.

**3D migration assay**

For 3D migration assays, 100 µl of Matrigel^TM^ (BD Bioscience) were polymerized in Transwell inserts. M1 macrophages were differentiated in the presence or absence of ibrutinib (0.3 µM) or DMSO as described before and then were seeded on the top of the matrix and the lower chamber of the Transwell filled with 250 µl RPMI 0.5 % FCS supplemented with CCL5(20 ng/ml).After 3 days, the percentage of migrating cells was determinedas the ratio of cells within thematrix to the total number of counted cells.

**Cytokine determination**

HD or CLL macrophages were pre-treated with vehicle (DMSO) or different doses of ibrutinib for 30 minutes and then stimulated with irradiated *Mtb* (MOI equivalent to 2), LPS (100 ng/ml) or Pam3CSK4 (100 ng/ml). Culture supernatants were collected 24 h later and TNF-α, IL-8 and IL-10 secretion were measured by ELISA.

Purified γδ T cells were pre-treated with vehicle (DMSO) or different doses of ibrutinib for 30 minutes and then stimulated with irradiated *Mtb* (MOI equivalent to 5). After 24 h IFN-ɣ production was measured in culture supernatants.

**Western blots**

Cell were harvested after different treatments and washed twice with cold PBS. Whole-cell lysates were prepared using loading buffer 1X- 5 % of β-mercaptoethanol and then boiled for 5 minutes. Protein extracts were separated on a standard 12% SDS–PAGE and transferred to PVDF membranes. Membranes were then blotted with primary antibodies, anti p-p65 or p-STAT1, followed by HRP-conjugated anti-rabbit IgG mAb. Specific bands were visualized by enhanced chemiluminiscence (ECL) method. The same membrane was blotted with mAb anti-β-actin followed by HRP-conjugated anti-mouse IgG to compare the total amount of protein in each sample. Densitometric measurements of specific bands were determinate by using ImageJ software (NIH).

**Bacterial growth in macrophages**

Macrophages seeded on glass coverslips within a 24-well tissue culture plate (Costar) at a density of 5 × 10^5^ cells/ml were infected with the red fluorescent protein (RFP) expressing M. tuberculosis CDC 1551 strain, gently provided by Dr. Fabiana Bigi (INTA, Castelar, Argentina), at a MOI of 5:1 during 2 h at 37°C. Then, extracellular bacteria were removed gently by washing with pre-warmed PBS, and cells were cultured in RPMI-1640 medium supplemented with 10% FBS for 48 h. The glass coverslips were fixed with PFA 4% and stained with BODIPY 493/503 (Life Technologies). Finally, slides were mounted and visualized with a FluoView FV1000 confocal microscope (Olympus, Tokyo, Japan) equipped with a Plapon 60X/NA1.42 objective, and then analyzed with the software ImageJ-Fiji. We measured the occupied area with RFP-M. tuberculosis (expressed as Raw Integrated Density) per cell in z-stacks from confocal laser scanning microscopy images. Individual cells were defined by BODIPY-stained cellular membranes which allow us to define the region of interests for quantification. For quantification 80-100 cells of random fields per condition were analyzed.

**Statistical analysis**

Statistical significance was determined using the non-parametric tests: Wilcoxon matched-pairs signed rank test to compare between two paired groups and Friedman test or Kruskal-Wallis followed by the Dunn’s post-test to compare three or more groups, matched or unmatched respectively. In all cases two-tailed tests were used and*p*<0.05 was considered statistically significant. Data were analyzed using the GraphPad Prism software version 7 and the GPower software.

**Supplementary Table 1**

**Clinical and biological features of CLL patients enrolled in the study.**

**Supplementary Figures**

**Supplementary Figure 1**

**Supplementary figure 1.** Macrophages were obtained by culturing monocytes from healthy donors (HD) for 5 days in RPMI 10 % FCS in the presence of M-CSF. **A.** Ibrutinib (Ibru) or the vehicle of the drug (DMSO) was added to the culture for 48 hs. The antineoplastic drug Lurbinectedin (Lur) was used as a positive control of cell death that was assessed by Flow Cytometry using the DNA dye 7-AAD. Bars represent mean ± SEM of the percentage of 7-AAD positives macrophages.**B-D.**Macrophages were stimulated with irradiated *Mtb* (MOI equivalent to 2), LPS (100 ng/ml) or Pam3CSK4 (100 ng/ml) in the presence or absence of ibrutinib (Ibru) and after 24 h TNF-α, IL-8 and IL-10 secretion was measured by ELISA. Bars represent mean ± SEM of TNF-α concentration in the culture supernatants. n=10, **p*˂0.05, Kruskal-Wallis test, followed by Dunn's multiple comparisons test.

**Supplementary Figure 2**

**Supplementary Figure 2. M1 and M2 macrophages´ surface markers.** To obtain the M1 profile monocytes from HD were cultured with GM-CSF (50 ng/ml) for 7 days and IFN-γ (10 ng/ml) was added for the last 2 days of culture.In order to obtain IL4-induced and IL-10 induced M2 macrophages, monocytes from HD were cultured with M-CSF (50 ng/ml) for 7 days and IL-4 (20 ng/ml) or IL-10 (10 ng/ml) was added for the last 2 days of culture. At day 7 macrophages were detached and stained with anti-CD14 PerCP/Cy5.5, anti-CD16 FITC, anti-CD86 PerCP/Cy5.5, anti-CD206 FITC, anti-CD163 PE or anti-HLA-DR FITC and analyzed by using a FACScan flow cytometer (BD Immunocytometry Systems, San Jose, CA, USA). Results are shown as the mean fluorescence intensity (MFI) normalized to the MFI of the isotype control.**p*˂0.05, Kruskal-Wallis test, followed by Dunn's multiple comparisons test.

**Supplementary Figure 3**

**Supplementary figure 3.** In order to obtain IL4-induced and IL-10 induced M2 macrophages, monocytes from HD were cultured with M-CSF (50 ng/ml) for 7 days and IL-4 (20 ng/ml) or IL-10 (10 ng/ml) was added for the last 2 days of culture. Ibrutinib (0.3 µM) was added to the culture 30 minutes before adding the cytokines. At day 7 macrophages were detached and stained with anti-CD14PerCP/Cy5.5, anti-CD16 FITC, anti-CD86 PerCP/Cy5.5, anti-CD206 FITC, anti-CD163 PE or anti-HLA-DR FITC and analyzed by using a FACScan flow cytometer (BD Immunocytometry Systems, San Jose, CA, USA). The results are shown as the mean ± SEM of MFI of each marker. n=8.

**Supplementary Figure 4**

**Supplementary figure 4.** Monocytes from healthy donors were differentiated into M1 macrophages in the presence or absence of ibrutinib as described before. At day 7 phosphorylation status of STAT1 was evaluated by western blot and bands on the immunoblots were quantified using the ImageJ software (NIH Image). Results are shown as the mean ± SEM of the STAT1/β-actinratio in arbitrary units (A.U.). n=5. N.S.: no statistically significant.Wilcoxon signed rank test.

**Supplementary Figure 5**

**Supplementary figure 5.** Purified monocytes from CLL patients were cultured with GM-CSF (50 ng/ml) for 7 days and IFN-γ (10 ng/ml) was added for the last 2 days of culture. Ibrutinib (0.3 µM) was added to the culture 30 minutes before adding IFN-γ. **A.** At day 7 macrophages were detached and stained withanti-CD16PE-Cy™5, anti-CD14 PE, anti-CD86 PE, anti-CD206 Alexa Fluor®488, anti-CD163PerCP/Cy5.5 and anti-HLA-DR FITC and expression level of the markers was assessed by flow cytometry. **p*˂0.05, Wilcoxon matched-pairs signed rank test. The results presented in this figure are part of an ongoing non-clinical Investigator Initiated Study (IIS) supported by Janssen. **B.** Glucose consumption and lactate production was evaluated in the culture supernatants of CLL-M1 macrophages differentiated in the presence or absence of ibrutinib. **p*˂0.05, Wilcoxon matched-pairs signed rank test.

**Supplementary figure 6**

**
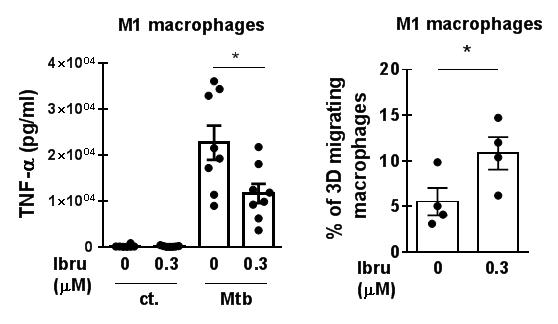
**

**Supplementary figure 6. Ibrutinib impairs TNF-α secretion and increases migration in matrigel on M1 polarized macrophages.** Monocytes from healthy donors were differentiated into M1 macrophages by culturing them 5 days with GM-CSF (50 ng/ml) and 2 additional days with GM-CSF plus IFN-ɣ (10 ng/ml). **A.** Then macrophages were pre-treated with ibrutinib (0.3 µM) for 30 minutes and then irradiated Mtb (MOI equivalent to 2) was added to the culture. After 24h TNF-α was evaluated in the supernatant by ELISA. Bars show the mean ± SEM. *p<0.05, Friedman test, Dunn's multiple comparisons test. **B.** After M1 polarization, macrophages were treated or not with ibrutinib (0.3 µM) and then seeded on top of a thick layer of Matrigel in the upper Transwell chamber. The lower chamber was filled with medium with CCL5. Migration was quantified after 3 days. Results are shown as the percentage of migrating macrophages. Bars show the mean ± SEM. *p<0.05, Mann Whitney test.

**Supplementary figure 7**


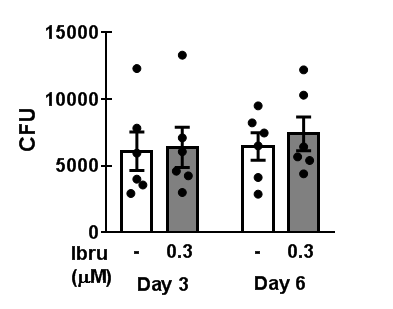


**Supplementary figure 7. Mtb growth on macrophages polarized in the presence or absence of ibrutinib.** Monocytes from healthy donors were differentiated into M1 macrophages in the presence or absence of ibrutinib as described before. Then macrophages were infected with viable Mtb (MOI= 2).At day 3 and 6 post infection macrophages were lysed and spread on plates containing BactoMiddlebrook 7H11 agar enriched with OADC. Colony-forming unit (CFU) enumeration was scored at day 21. Results are shown as the mean ± SEM of CFU. n= 6.
